# Development and preliminary validation of the Inadequate Child Care Scale in China

**DOI:** 10.1371/journal.pone.0344734

**Published:** 2026-03-11

**Authors:** Zhang Jiayuan, Yang Jinwei, Zhou Yuqiu

**Affiliations:** 1 Department of Nursing, Harbin Medical University, Daqing, China; 2 Key Laboratory of Basic Research and Health Management on Chronic Diseases in Heilongjiang Province, Daqing, Heilongjiang, China; 3 Department of Medical, Huzhou University, Huzhou, China; Transilvania University of Brasov: Universitatea Transilvania din Brasov, ROMANIA

## Abstract

**Background:**

Socio-economic changes and evolving family structures have created unique caregiving challenges in China, highlighting the need for tools to systematically measure child care deficits. This study aims to develop and preliminarily validate the Inadequate Child Care Scale (ICCS), a culturally relevant instrument designed to measure deficits in child care specific to the Chinese context.

**Methods:**

The development and validation of the Inadequate Child Care Scale (ICCS) were conducted through a three-phase process. Phase One involved the generation of an initial item pool informed by a prior grounded theory study. Phase Two included nationwide data collection via a structured survey administered to participants across diverse regions in China. Phase Three focused on evaluating the psychometric properties of the ICCS using Exploratory Factor Analysis (EFA; n = 468) and Confirmatory Factor Analysis (CFA; n = 702), with an emphasis on assessing convergent validity, discriminant validity, and composite reliability.

**Results:**

The initial item pool comprised 32 items, 30 of which were retained following expert evaluation for content validity. EFA revealed a four-factor structure underlying the scale: Inadequate Daily Life Care, Inadequate Psychological and Emotional Care, Inadequate Safety Care, and Inadequate Educational Care, encompassing 30 items in total. CFA supported the factorial validity of the ICCS, yielding favorable model fit indices (GFI = 0.929, CFI = 0.977, TLI = 0.975, RMSEA = 0.038, χ²/df = 2.025). The scale demonstrated strong internal consistency (Cronbach’s alpha > 0.80) and acceptable test-retest reliability.

**Conclusion:**

The ICCS is a psychometrically sound tool for assessing child care deficits in China. Its development fills a critical gap in child care research and provides a foundation for targeted interventions and policy reforms. Future studies should refine the scale further and explore its applications in broader caregiving contexts.

## 1. Introduction

Child care plays a pivotal role in shaping children’s overall development, profoundly influencing their physical health, emotional well-being, social skills, and cognitive growth [1]. Adequate and responsive care during childhood lays the foundation for lifelong resilience, adaptability, and the ability to thrive in various domains [2]. Conversely, deficiencies in child care can hinder developmental milestones, compromising the child’s potential and contributing to long-term adverse outcomes [3]. Ensuring appropriate caregiving is thus a cornerstone of promoting children’s rights and well-being globally [4].

Despite its importance, challenges in providing consistent and sufficient child care persist both globally and in China [5,6]. Internationally, child neglect is recognized as one of the most common forms of child maltreatment, characterized by the failure to meet a child’s basic needs, such as nutrition, healthcare, supervision, and emotional support [7,8]. Reports indicate that millions of children are affected by neglect each year, leading to developmental delays, behavioral issues, and mental health challenges [9,10]. In China, unique socio-cultural and economic factors have further complicated caregiving practices. Rapid urbanization, rural-urban migration, and evolving family structures, such as the rise of “left-behind children” and increased reliance on elderly caregivers, have introduced new caregiving challenges [11,12]. While child neglect has been extensively studied and measured through various tools [13,14], these scales often focus on broader global definitions of neglect, which may not fully capture the nuanced caregiving gaps specific to the Chinese context.

The concept of inadequate child care has emerged as a distinct construct, differing from child neglect in that it often results from systemic, contextual, or resource-related challenges rather than intentional omissions. This perspective is particularly relevant in China, where caregiving practices are shaped by structural and socio-economic factors. Existing scholars, using grounded theory, have defined inadequate child care in Chinese families as the failure of caregivers to provide adequate care for children across multiple dimensions, including daily living, emotional, psychological, safety, and educational aspects, due to the influence of internal family factors and external environmental factors [15]. Despite its importance, however, no culturally adapted tools currently exist to systematically assess the prevalence and impact of inadequate child care.

To bridge this gap, the present study aims to develop and preliminarily validate the Inadequate Child Care Scale (ICCS), a measurement tool tailored to the Chinese context. Building on a grounded theoretical framework, this study will systematically define the dimensions of child care deficit and evaluate the psychometric properties of the scale. By addressing the unique caregiving challenges faced in China, this work seeks to provide a foundational tool for research, intervention, and policymaking to improve caregiving practices and outcomes for children.

### 1.1. Purpose of the study

The development and validation of the Inadequate Child Care Scale (ICCS) aim to address several critical needs in the field of child care and developmental research. Firstly, there is a significant gap in the existing literature regarding comprehensive and psychometrically sound tools specifically designed to assess inadequate in child care, particularly in the Chinese context. Currently available instruments, such as the Child Neglect Scale (CNS) and Mother-Child Neglect Scale (MCNS), primarily focus on assessing neglect as a form of overt or intentional failure to meet a child’s basic needs, often framed within broad, generalized contexts [13,14]. While these tools contribute valuable insights into neglect, they do not fully capture the systemic, cultural, and contextual caregiving challenges unique to China, such as those stemming from socio-economic disparities, rural-urban migration, or the shifting family dynamics caused by multi-generational caregiving. In contrast, the Inadequate Child Care Scale (ICCS) is specifically designed to address these nuanced caregiving gaps, emphasizing deficits in child care that may arise from structural or contextual barriers rather than intentional neglect, thereby providing a more comprehensive and culturally relevant assessment of caregiving challenges in the Chinese context.

Secondly, a validated measure of child care is essential for advancing understanding of the factors contributing to caregiving challenges. By systematically assessing these deficits, researchers and practitioners can identify key elements that influence child care quality, including parental resources, social support, and community infrastructure. Insights gained from using the ICCS can inform targeted interventions and policy decisions aimed at improving child care practices and addressing the underlying causes of inadequate child care.

The ICCS also holds practical relevance for a wide range of stakeholders, including educators, child welfare professionals, policymakers, and caregivers. Child welfare practitioners can use the ICCS to identify children and families at risk of experiencing care deficits and to design tailored support programs. Policymakers can leverage data from the ICCS to inform strategies for improving caregiving resources and infrastructure. Additionally, longitudinal use of the ICCS allows practitioners to monitor changes in caregiving quality over time and evaluate the effectiveness of interventions aimed at reducing inadequate child care.

Overall, this study highlights the importance of addressing inadequate child care as distinct from neglect and emphasizes the need for a culturally relevant tool to measure these caregiving challenges. The development and validation of the ICCS fill a critical gap in the literature, providing a valuable resource for enhancing child care quality, promoting child well-being, and supporting evidence-based interventions and policies.

## 2. Methods

### 2.1. Study design

This study used a cross-sectional design, following DeVellis’ guidelines for scale development [16].The process included item generation, expert evaluation, and a validation study. The recruitment period for this study started on 30/9/2024 and ended on 30/11/2024.

### 2.2. Ethical considerations

The research protocol was reviewed and approved by the Ethics Board of Harbin Medical University- Daqing Campus. Participants received detailed information about the objectives, methods, potential risks, and benefits. Informed consent was obtained through signed forms. All methods were carried out in accordance with relevant guidelines and regulations.

### 2.3. Sample size calculation

Following the recommendations for exploratory factor analysis (EFA), we determined our sample size based on the subject-to-item ratio. Comrey and Lee suggest that samples of 300 are good and 500 are very good for factor analysis [17]. Additionally, the commonly accepted guideline recommends a minimum ratio of 5:1 (participants to items) for EFA [18]. With the initial ICCS containing 32 items, a minimum of 160 participants (5 × 32) was necessary. However, to ensure greater reliability, statistical power, and generalizability of the findings, we recruited 468 participants for the first phase of the study, which exceeds the recommended standards.

For confirmatory factor analysis (CFA), which requires an independent sample of similar size [19], 702 participants were included in the second phase of the study. This exceeded the minimum required sample size and ensured robust validation of the scale’s psychometric properties. By recruiting larger samples for both phases, this study aimed to achieve more reliable and generalizable results.

### 2.4. Eligibility criteria

Participants were family caregivers of children aged 6–16 years who met the following criteria:

① The parent or primary caregiver of a child within the specified age range.
② Living with the child for at least six months prior to the study.
③ Able to provide informed consent and participate in the study.
④ Capable of reading and understanding Chinese.

Parents of children with severe physical or cognitive disabilities, or those unable to communicate effectively were excluded to ensure consistent and reliable responses related to typical caregiving practices.

### 2.5. Research process

#### Stage one: Items generation.

The initial item pool for the Inadequate Child Care Scale (ICCS) was developed based on a prior qualitative study using grounded theory. This study identified four core dimensions of inadequate child care through coding and thematic analysis, which were further refined through a comprehensive review of relevant literature and in-depth interviews with caregivers and child care experts.

An initial draft of the scale was constructed, consisting of 56 items designed to measure inadequate child care across these four dimensions. The items underwent rigorous review and revision through iterative consultations with experts in child development, caregiving practices, and psychometrics, as well as interviews with parents, ensuring clarity, cultural relevance, and content validity. The semantic accuracy and practical relevance of each item were critically evaluated during this process.

A 7-point Likert scale was selected for the ICCS to measure the degree of perceived inadequate child care, ranging from 1 (“strongly disagree”) to 7 (“strongly agree”). This scale was chosen for several reasons. First, it provides respondents with a nuanced way to express their perceptions, offering greater precision in capturing variations in responses [20]. Second, the 7-point Likert scale improves data accuracy by minimizing central tendency bias and offering a broader range of choices for respondents. Third, it allows for more detailed differentiation in attitudes or perceptions, which is critical for identifying specific inadequate care [21].

Following the finalization of the initial scale, a structured questionnaire was designed, and data were collected through a pilot study to evaluate the scale’s feasibility and psychometric properties.

#### Stage two: Expert consultation.

The initial item pool for the ICCS underwent two rounds of expert consultation to ensure content validity and clarity. The consultation form consisted of two parts:

Evaluation Form for the ICCS: Experts were asked to evaluate the relevance of each secondary indicator to its corresponding primary indicator using a 4-point scale (not relevant, weakly relevant, moderately relevant, highly relevant). Experts also assessed the importance of each secondary indicator to the overall theme using a 5-point Likert scale (very unimportant, unimportant, neutral, important, very important).

Expert Profile Survey: This section gathered information about the experts, including their basic demographic details, the basis of their judgments, and their familiarity with the subject matter.

The consultation process was conducted electronically via email, allowing for convenient distribution and collection. Based on the feedback from experts, revisions were made to the content and wording of the items. Items that did not meet the following criteria were removed: ① Item content validity index (I-CVI) < 0.80; ② Mean importance score < 4 [22]. Additionally, items identified by experts as repetitive or unclear were revised or removed following discussions within the research team. After two rounds of consultation and refinement, the revised initial version of the ICCS was finalized with 32 items.

#### Stage three: Administration of ICCS.

Before the validation study, a pilot test was conducted to assess the reliability and clarity of the questionnaire. The pilot study aimed to evaluate participants’ understanding of the items, the difficulty level of the questions, and the time required to complete the questionnaire, ensuring that potential issues could be identified and resolved prior to the main study. The results indicated that participants demonstrated a good understanding of all items, with no significant difficulties reported. The average time required to complete the questionnaire was approximately 8 minutes, confirming its feasibility for use in the larger validation study.

The finalized ICCS was tested on a large sample of family caregivers to evaluate its psychometric properties, including reliability and validity. Data were collected through both online surveys and paper-based questionnaires, with participant demographics (e.g., age, gender, caregiving characteristics) recorded for analysis. The validation study included two phases: recruiting participants for Exploratory Factor Analysis (n = 468) and Confirmatory Factor Analysis (n = 702).

### 2.6. Data analysis

Data were double-entered and cross-checked using EpiData to ensure accuracy. SPSS 26.0 was employed for Exploratory Factor Analysis (EFA), utilizing methods such as correlation analysis, critical ratio method, homogeneity testing, and factor analysis for item selection [23,24]. AMOS 23.0 was used for Confirmatory Factor Analysis (CFA) to validate the factor structure of the scale [25]. A significance level of P < 0.05 was considered statistically significant for all analyses.

## 3. Results

### 3.1. Demographic statistics

For the Exploratory Factor Analysis (EFA), a total of 468 questionnaires were analyzed. Among the caregivers, 346 were parents, 104 were grandparents, and 18 were other caregivers. For the Confirmatory Factor Analysis (CFA), 702 questionnaires were collected. The caregivers included 517 parents, 164 grandparents, and 21 other caregivers.

### 3.2. Pilot study data analysis results (N=468)

The pilot study included 468 valid responses to assess the preliminary performance of the ICCS. Key findings from the analysis are as follows:

#### 3.2.1. Reliability.

The scale consisted of 32 items across four latent variables, with an overall Cronbach’s alpha value of 0.940, indicating excellent reliability. The reliability coefficients for the four dimensions were as follows: Dimension 1 (0.941), Dimension 2 (0.952), Dimension 3 (0.937), and Dimension 4 (0.947). All coefficients exceeded the commonly accepted threshold of 0.7, demonstrating that the questionnaire possesses good reliability and internal consistency.

Additionally, the corrected item-total correlation (CITC) values for all items were above the threshold of 0.5, indicating that the items were well-aligned with their respective latent variables and supported the overall reliability of the questionnaire. An item deletion analysis was performed, where each item was removed individually to assess its impact on the overall Cronbach’s alpha. The results revealed that deleting Item 19 and Item 32 improved the overall reliability of the scale. Conversely, removing any other items did not increase the alpha value, confirming their strong reliability. Therefore, Item 19 and Item 32 were excluded, and the remaining items demonstrated high reliability and consistency. Seen in Table 1.

**Table 1:** Reliability test of each variable in the questionnaire(n = 468).

Variable	Item	CITC	Deleted item Klonbach Alpha	Klonbach Alpha
Dimension 1	Item 1	0.806	0.932	0.941
	Item 2	0.786	0.933	
	Item 3	0.856	0.929	
	Item 4	0.750	0.936	
	Item 5	0.788	0.933	
	Item 6	0.788	0.933	
	Item 7	0.778	0.934	
	Item 8	0.767	0.934	
Dimension 2	Item 9	0.838	0.945	0.952
	Item 10	0.835	0.945	
	Item 11	0.812	0.946	
	Item 12	0.806	0.947	
	Item 13	0.796	0.947	
	Item 14	0.834	0.945	
	Item 15	0.827	0.945	
	Item 16	0.830	0.946	
Dimension 3	Item 17	0.879	0.919	0.937
	Item 18	0.784	0.928	
	Item 19	0.580	0.946	
	Item 20	0.831	0.924	
	Item 21	0.851	0.922	
	Item 22	0.847	0.922	
	Item 23	0.795	0.927	
Dimension 4	Item 24	0.833	0.939	0.947
	Item 25	0.810	0.940	
	Item 26	0.876	0.936	
	Item 27	0.827	0.939	
	Item 28	0.826	0.939	
	Item 29	0.835	0.939	
	Item 30	0.834	0.939	
	Item 31	0.781	0.942	
	Item 32	0.540	0.955	
Overall reliability of the scale				0.940

#### 3.2.2. Validity.

The exploratory factor analysis (EFA) results demonstrated that the questionnaire is suitable for factor analysis. The KMO value was 0.956, exceeding the threshold of 0.70, indicating the adequacy of the sample for factor analysis. Bartlett’s test of sphericity yielded a chi-square value of 12717.811, with a significance level of P < 0.01, confirming the appropriateness of conducting factor analysis and supporting the questionnaire’s strong structural validity.

Using the Principal Factor Analysis method with orthogonal rotation via the maximum variance method, 30 items were grouped into four factors with eigenvalues greater than 1. These four factors accounted for 75.233% of the total variance, surpassing the commonly accepted standard of 60%. Factor loadings for all items were above 0.5, and no items exhibited high loadings on multiple factors. Furthermore, the items for each dimension aggregated according to theoretical expectations, confirming the scale’s strong content validity. Based on reliability and validity analyses, Item 19 and Item 32 were identified for removal to optimize the scale. The remaining items were well-structured and valid, forming a refined questionnaire ready for formal research in subsequent phases. Seen in Table 2.

**Table 2:** Results of factor analysis (n = 468).

Item	Factor			
	1	2	3	4
Item 1			0.802	
Item 2			0.793	
Item 3			0.826	
Item 4			0.735	
Item 5			0.785	
Item 6			0.82	
Item 7			0.778	
Item 8			0.779	
Item 9		0.809		
Item 10		0.804		
Item 11		0.803		
Item 12		0.790		
Item 13		0.780		
Item 14		0.795		
Item 15		0.796		
Item 16		0.802		
Item 17				0.895
Item 18				0.829
Item 20				0.870
Item 21				0.888
Item 22				0.887
Item 23				0.851
Item 24	0.856			
Item 25	0.844			
Item 26	0.893			
Item 27	0.854			
Item 28	0.867			
Item 29	0.855			
Item 30	0.871			
Item 31	0.830			
**Eigenvalue**	11.368	5.290	3.857	2.055
**Interpretation variance** %	37.894	17.632	12.857	6.851
**Total explanatory variance** %	75.233			

### 3.3. Large sample test results (N=702)

The study included 702 valid responses to evaluate the psychometric properties and performance of the ICCS. Key findings are summarized as follows:

#### 3.3.1. Reliability.

The ICCS consisted of 30 measurement items across four latent variables. The overall reliability of the scale, measured by Cronbach’s alpha, was 0.936, indicating excellent internal consistency. The reliability coefficients for the four dimensions were as follows: Dimension 1 (0.941), Dimension 2 (0.943), Dimension 3 (0.945), and Dimension 4 (0.949). All coefficients exceeded the commonly accepted threshold of 0.7, demonstrating that the questionnaire possesses strong reliability and internal consistency.

Additionally, the corrected item-total correlation (CITC) values for all items were above 0.5, confirming that the items were well-aligned with their respective latent variables and that the overall scale exhibited good reliability. An item deletion analysis was conducted by removing one item at a time to assess its impact on the overall Cronbach’s alpha. The results showed that deleting any single item did not lead to an increase in the overall reliability, indicating that all items were appropriately designed and contributed to the scale’s consistency. These findings confirm that the ICCS has excellent reliability and internal coherence. Seen in Table 3.

**Table 3:** Reliability test of each variable in the questionnaire(n = 702).

Variable	Item	CITC	Deleted item Klonbach Alpha	Klonbach Alpha
Dimension 1	Item 1	0.790	0.933	0.941
	Item 2	0.778	0.934	
	Item 3	0.850	0.929	
	Item 4	0.775	0.934	
	Item 5	0.816	0.931	
	Item 6	0.725	0.938	
	Item 7	0.799	0.933	
	Item 8	0.789	0.933	
Dimension 2	Item 9	0.804	0.935	0.943
	Item 10	0.810	0.934	
	Item 11	0.795	0.935	
	Item 12	0.777	0.937	
	Item 13	0.771	0.937	
	Item 14	0.801	0.935	
	Item 15	0.805	0.935	
	Item 16	0.795	0.936	
Dimension 3	Item 17	0.875	0.930	0.945
	Item 18	0.790	0.940	
	Item 19	0.825	0.936	
	Item 20	0.853	0.932	
	Item 21	0.857	0.932	
	Item 22	0.800	0.939	
Dimension 4	Item 23	0.815	0.942	0.949
	Item 24	0.790	0.943	
	Item 25	0.865	0.938	
	Item 26	0.820	0.941	
	Item 27	0.806	0.942	
	Item 28	0.812	0.942	
	Item 29	0.831	0.941	
	Item 30	0.763	0.945	
Overall reliability of the scale				0.936

#### 3.3.2. Validity.

The exploratory factor analysis (EFA) demonstrated that the ICCS is suitable for factor analysis. The KMO value was 0.893, exceeding the threshold of 0.70, indicating sampling adequacy for factor analysis. Bartlett’s test of sphericity yielded a chi-square value of 3233.039, with a significance level of P < 0.01, confirming that the data were appropriate for factor analysis and supporting the strong structural validity of the scale.

Using Principal Factor Analysis with orthogonal rotation via the maximum variance method, 30 items were grouped into four factors with eigenvalues greater than 1. These four factors accounted for 73.627% of the total variance, which exceeds the commonly accepted standard of 60%, indicating strong validity. All items had factor loadings greater than 0.5, and no items displayed high cross-loadings on multiple factors. Additionally, items for each dimension aggregated according to theoretical expectations, confirming that the scale has excellent content validity. Seen in Table 4.

**Table 4:** Results of factor analysis (n = 702).

Item	Factor			
	1	2	3	4
Item 1		0.772		
Item 2		0.781		
Item 3		0.818		
Item 4		0.791		
Item 5		0.815		
Item 6		0.744		
Item 7		0.820		
Item 8		0.808		
Item 9			0.801	
Item 10			0.799	
Item 11			0.788	
Item 12			0.785	
Item 13			0.764	
Item 14			0.786	
Item 15			0.797	
Item 16			0.781	
Item 17				0.896
Item 18				0.833
Item 19				0.869
Item 20				0.893
Item 21				0.890
Item 22				0.855
Item 23	0.839			
Item 24	0.833			
Item 25	0.890			
Item 26	0.851			
Item 27	0.849			
Item 28	0.837			
Item 29	0.859			
Item 30	0.812			
**Eigenvalue**	10.822	5.034	4.034	2.198
**Interpretation variance** %	36.074	16.779	13.445	7.328
**Total explanatory variance** %	73.627			

#### 3.3.3. Validity test: Confirmatory factor analysis using AMOS.

**Fig 1:**
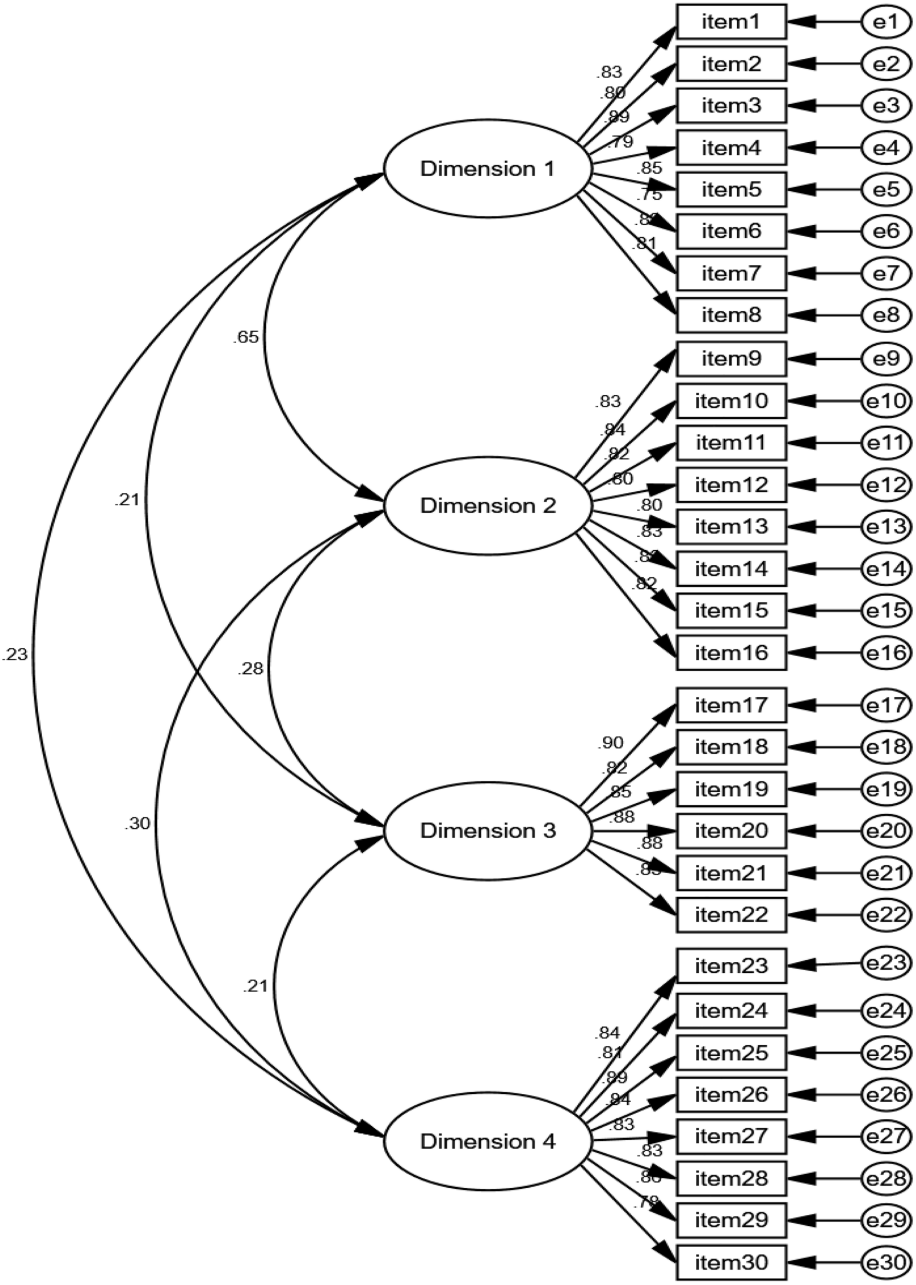
Model of CFA for ICCS.

In this study, confirmatory factor analysis (CFA) was conducted using AMOS software. Model fit was evaluated comprehensively using several fit indices: goodness-of-fit indices (χ², df, and χ²/df < 3), relative fit indices (TLI and CFI > 0.9), and absolute fit indices (RMSEA< 0.08). The final model (Fig 1), showed a good model fit for ICCS. To determine the optimal fit, four competing models were designed and compared. The comparative results of model fit indices are presented in Table 5. As shown in Table 5, the four-factor model demonstrated the best fit, with all fit indices meeting the specified criteria. The factor loadings for each indicator within their respective dimensions are presented in Table 6. The standardized factor loadings ranged from 0.746 to 0.905, and all items passed the t-test (*p* < 0.00), confirming the construct validity of the four dimensions. Furthermore, a second-order factor analysis was performed, and the second-order factor model exhibited excellent fit indices, indicating the robustness of the proposed model.

**Table 5:** Comparison of fitting indexes of each model.

Model	Factor	X²	df	X²/df	TLI	CFI	RMSEA
Four-factor model	A, B, C, D	808.028	399	2.025	0.975	0.977	0.038
Three-factor model	A + B, C, D	2929.488	402	7.287	0.847	0.859	0.095
Two-factor model	A + B + C, D	6436.146	404	15.931	0.636	0.662	0.146
Single factor model	A + B + C + D	10745.068	405	26.531	0.378	0.421	0.191
Second-order Factor model		817.450	401	2.039	0.975	0.977	0.038

Note: A-Dimension 1, B- Dimension 2, C- dimension 3, D- dimension 4.

**Table 6:** Standardized factor loads.

Variable	Item	Standardize factor loads	S.E.	*t*	*P*
Dimension 1	Item 1	0.825			
	Item 2	0.804	0.034	25.368	<0.000
	Item 3	0.886	0.039	29.536	<0.000
	Item 4	0.794	0.036	24.888	<0.000
	Item 5	0.846	0.038	27.417	<0.000
	Item 6	0.746	0.033	22.750	<0.000
	Item 7	0.822	0.038	26.225	<0.000
	Item 8	0.813	0.036	25.779	<0.000
Dimension 2	Item 9	0.830			
	Item 10	0.837	0.036	27.216	<0.000
	Item 11	0.821	0.035	26.441	<0.000
	Item 12	0.803	0.035	25.520	<0.000
	Item 13	0.798	0.035	25.302	<0.000
	Item 14	0.828	0.036	26.794	<0.000
	Item 15	0.832	0.035	26.962	<0.000
	Item 16	0.823	0.033	26.508	<0.000
Dimension 3	Item 17	0.905			
	Item 18	0.820	0.028	30.222	<0.000
	Item 19	0.853	0.026	32.895	<0.000
	Item 20	0.881	0.026	35.476	<0.000
	Item 21	0.884	0.025	35.677	<0.000
	Item 22	0.828	0.026	30.868	<0.000
Dimension 4	Item 23	0.840			
	Item 24	0.813	0.035	26.630	<0.000
	Item 25	0.892	0.041	31.124	<0.000
	Item 26	0.844	0.039	28.313	<0.000
	Item 27	0.832	0.039	27.622	<0.000
	Item 28	0.833	0.039	27.662	<0.000
	Item 29	0.856	0.036	28.994	<0.000
	Item 30	0.783	0.037	25.079	<0.000

#### 3.3.4. Convergent validity and discriminant validity.

Based on the confirmatory factor analysis (CFA), this study calculated the composite reliability (CR) and the average variance extracted (AVE) to evaluate convergent validity. Since testing only one of these indicators is insufficient to verify convergent validity, this study adopted the thresholds of CR > 0.8 and AVE > 0.5. In addition, two methods were employed to test the discriminant validity of the scale. The results are shown in Table 7. As can be seen from Table 7, the CR and AVE values for Dimension 1, Dimension 2, Dimension 3, and Dimension 4 all meet the required standards, indicating good convergent validity for all dimensions. Furthermore, the standardized correlation coefficients between each pair of dimensions are smaller than the square root of the AVE values for their respective dimensions. Additionally, the model fit indices from the earlier confirmatory factor analysis demonstrated that the four-factor model achieved better fit than alternative models. These results collectively confirm that the scale exhibits ideal discriminant validity among the dimensions.

**Table 7:** Results of aggregate validity and discriminative validity.

	CR	AVE	Dimension 1	Dimension 2	Dimension 3	Dimension 4
Dimension 1	0.942	0.669	**0.818**			
Dimension 2	0.943	0.675	0.612	**0.822**		
Dimension 3	0.946	0.744	0.2	0.26	**0.862**	
Dimension 4	0.949	0.701	0.216	0.279	0.202	**0.837**

## 4. Discussion

This study developed and validated a psychometrically sound Inadequate Child Care Scale (ICCS) to assess parental caregiving deficits for children aged 6–16 in China. The results of exploratory factor analysis (EFA) and confirmatory factor analysis (CFA) demonstrated that the 30-item ICCS consists of four interrelated but distinct subscales, which evaluate different dimensions of inadequate child care: inadequate in daily care, inadequate in psychological and emotional care deficit, inadequate in safety care, and inadequate in educational care. All four subscales showed excellent internal consistency, with Cronbach’s alpha coefficients exceeding 0.8, confirming the reliability of the ICCS. These findings indicate that the ICCS is a reliable and valid instrument for measuring caregiving deficits across multiple dimensions, providing a comprehensive tool for understanding the parental caregiving situation for children in China.

The development of the ICCS began with a grounded theory approach to conceptualize the key dimensions of child care deficits. This process resulted in an initial pool of 56 items designed to comprehensively capture multiple facets of inadequate child care. To ensure content validity and clarity, a two-round Delphi expert consultation was conducted, during which 24 items were eliminated due to redundancy, ambiguity, or irrelevance, leaving 32 items for further analysis. Subsequently, Exploratory Factor Analysis (EFA) revealed a four-factor structure, representing distinct yet interrelated dimensions of inadequate child care that impact children’s well-being. During the item analysis stage, it was found that the overall Cronbach’s alpha coefficient increased when item 19 and item 32 were removed, suggesting these items negatively affected the scale’s internal consistency. Consequently, both items were excluded, resulting in a refined 30-item scale. To further validate the factor structure, Confirmatory Factor Analysis (CFA) was performed. The scale demonstrated strong psychometric properties, including good internal consistency (Cronbach’s α > 0.8), as well as evidence for convergent validity and discriminant validity.

The dimensions of the ICCS are closely aligned with established psychological and caregiving theories, particularly Maslow’s hierarchy of needs, which progresses from physiological needs to safety, belonging, and self-actualization [26,27]. For example, the inadequate daily care dimension corresponds to physiological needs, addressing children’s access to proper nutrition, hygiene, and physical well-being, while the inadequate in psychological and emotional care reflects elements of belonging and esteem needs, highlighting the critical role of emotional support, affection, and nurturing relationships for a child’s psychological health. This alignment demonstrates the comprehensiveness and scientific rigor of the ICCS, distinguishing it from unidimensional neglect measures that often focus on physical or emotional neglect alone [28]. Supporting this multidimensional approach, findings from previous research further underscore the need for holistic caregiving assessments. For instance, studies have shown that safety deficits, such as a lack of parental supervision or exposure to hazardous environments, can significantly increase the risk of childhood injuries and long-term psychological trauma [29,30]. Similarly, research on daily care deficits emphasizes the impact of inadequate routines, poor nutrition, and hygiene neglect on children’s physical health, cognitive development, and overall well-being [31–33]. These findings underscore the comprehensive and representative nature of the ICCS dimensions, which effectively capture the diverse inadequate caregiving impacting children’s development.

Moreover, the ICCS not only complements these studies by addressing universal caregiving needs but also extends existing frameworks by capturing culturally specific caregiving deficits. The items included in the ICCS were carefully designed to address current caregiving practices and challenges. For instance, in the inadequate educational care dimension, the scale incorporates issues such as “harsh parenting” and “overemphasis on academic achievement”—problems particularly prominent in Chinese parenting culture [34,35]. Traditional parenting ideologies, such as punitive educational practices and the singular focus on academic success, often result in neglecting a child’s psychological well-being and overall development [36,37]. By identifying inadequate educational care, the ICCS provides a culturally tailored tool to measure this phenomenon and highlights the need for balanced parenting approaches that prioritize children’ s holistic growth. Similarly, in the inadequate daily care dimension, the ICCS reflects changing family dynamics and parenting priorities in the context of economic and societal advancement. Historically, caregiving assessments primarily focused on basic needs such as adequate food and shelter [38]. However, with improved living conditions, modern parenting has shifted towards scientific and evidence-based caregiving, emphasizing aspects such as balanced nutrition, physical activity, and healthy habits [39–41]. Items addressing concerns like picky eating, lack of exercise, and irregular routines resonate with these contemporary caregiving trends, ensuring that the scale remains highly relevant for today’ s parenting realities.

By integrating universal theories with contemporary and cultural caregiving issues, the ICCS serves as a scientifically grounded and contextually relevant tool for evaluating child care deficits. This multidimensional and adaptive approach ensures that the ICCS can effectively capture both traditional and modern caregiving challenges, providing researchers, practitioners, and policymakers with a robust instrument to identify caregiving gaps and promote improved child welfare outcomes.

### 4.1. Strengths and limitations

This study develops and validates the ICCS, providing a comprehensive and scientifically grounded tool for assessing multidimensional inadequate caregiving with strong cultural relevance and psychometric validity. However, the study has limitations. The sample was drawn from specific regions in China, which may limit the generalizability of the findings to other populations or cultural contexts. Additionally, the reliance on parental self-reporting introduces potential biases, such as social desirability or inaccuracies in recalling caregiving practices. While the ICCS effectively captures inadequate caregiving, its predictive validity remains to be explored.

### 4.2. Implications for future practice and research

The ICCS has significant implications for both researchers and practitioners. For practitioners, the scale offers a reliable tool to identify specific inadequate caregiving across several key domains: daily care, psychological and emotional care, safety care, and educational care. This allows for targeted interventions, such as parental education programs, mental health support, and safety awareness campaigns, which can address specific caregiving gaps and improve the overall well-being of children [42]. For policymakers, the ICCS can inform the development of policies and programs that support families, particularly those with identified inadequate caregiving in critical areas.

For future research, the ICCS provides valuable opportunities to investigate the relationship between inadequate caregiving and child developmental outcomes. Longitudinal studies would be especially beneficial in examining how deficits in various caregiving domains impact children’s mental health, educational achievement, and overall well-being over time. Furthermore, cross-cultural research could assess the scale’s applicability in diverse cultural and socioeconomic contexts, providing comparative insights into global caregiving practices. Incorporating multiple perspectives, such as children’s self-reports and teacher evaluations, could enhance the understanding of caregiving deficits and offer a more comprehensive view of child development.

## 5. Conclusion

In conclusion, the ICCS represents a significant contribution to understanding and addressing inadequate caregiving in China (Table 8). Through its comprehensive structure, strong psychometric validation, and cultural relevance, the ICCS provides a reliable and valid tool for assessing the various dimensions of caregiving that affect children’s well-being. By integrating universal caregiving theories with culturally specific challenges, the scale ensures a holistic approach to evaluating inadequate child care. The ICCS not only advances scientific knowledge of inadequate caregiving but also has practical implications for improving child welfare, guiding interventions, and informing policy. Future research and broader application of the ICCS can further enhance its utility, ensuring that it continues to be an effective instrument for addressing caregiving gaps and promoting healthier developmental outcomes for children.

**Table 8:** The Inadequate Child Care Scale (ICCS).

Item	Content
Item 1	I prepare three meals a day for my child on time.
Item 2	I restrain my child from being picky with food and eating too many snacks.
Item 3	I remind my child to go to bed on time.
Item 4	I encourage my child to exercise regularly.
Item 5	I pay attention to my child’s physical health and growth.
Item 6	I can promptly notice when my child feels unwell and take care of them carefully.
Item 7	I help my child with personal hygiene management.
Item 8	I provide my child with a clean living environment.
Item 9	I spend time chatting and playing with my child.
Item 10	When I’m with my child, I rarely get busy with work or playing on my phone.
Item 11	When my child is feeling down, I provide effective comfort.
Item 12	I often express my love for my child through words or hugs.
Item 13	I pay attention to my child’s emotional and behavioral changes.
Item 14	I can understand my child’s needs and feelings.
Item 15	I often support my child’s growth and progress through praise and encouragement.
Item 16	When my child faces difficulties, I am able to offer support and help.
Item 17	I eliminate safety hazards in the home.
Item 18	I pay attention to my child’s outdoor play activities.
Item 19	I teach my child to recognize safety issues such as bullying and privacy violations.
Item 20	I teach my child how to handle situations when they are bullied.
Item 21	I guide my child to avoid dangerous activities instead of prohibiting or commanding them.
Item 22	I teach my child how to handle emergencies (such as fire, earthquake, getting lost, or injury).
Item 23	I correct my child’s bad behavior and habits.
Item 24	I set an example for my child through my own behavior.
Item 25	I understand my child’s interests, hobbies, and strengths.
Item 26	I encourage my child to participate in competitions, gatherings, and other social activities.
Item 27	I guide my child in expressing their emotions.
Item 28	When my child faces stress or setbacks, I help them face it positively.
Item 29	I rarely use harsh language or corporal punishment to educate my child.
Item 30	I tend to guide my child to correct mistakes, rather than criticize them.

Note: Inadequate in Daily Life Care = 1–8;Inadequate in Psychological and Emotional Care = 9–16, Inadequate in Safety Care = 17–22, and Inadequate in Educational Care = 23–30
